# Rapid, Recurrent Unilateral Pleural Effusion Presenting as Cryptococcus Infection in a Non-immunocompromised Host

**DOI:** 10.7759/cureus.115732

**Published:** 2026-09-03

**Authors:** Malia Gacutan, Khadija Bhatti, Ashley Jordan, Alexis R Aiman, Brijesh B Patel

**Affiliations:** 1 Medicine, Edward Via College of Osteopathic Medicine, Blacksburg, USA; 2 Internal Medicine, Carilion Clinic, Roanoke, USA; 3 Critical Care Medicine, Carilion Clinic, Roanoke, USA; 4 Pulmonary and Critical Care Medicine, Carilion Clinic, Christiansburg, USA; 5 Internal Medicine/Pulmonary and Critical Care, LewisGale Medical Center, Salem, USA

**Keywords:** cryptococcus neoformans (c. neoformans), diagnostic process, immuno-competent, pulmonary cryptococcal infection, recurrent pleural effusions

## Abstract

*Cryptococcus neoformans* infection presenting as a rapidly reaccumulating unilateral pleural effusion in a non-immunocompromised host is a rare presentation of pulmonary cryptococcal disease. We present a rare case of recurrent unilateral pleural effusion as the initial presentation of cryptococcal disease in an immunocompetent patient. An 85-year-old male with a past medical history of hypertension, hyperlipidemia, diabetes mellitus, chronic kidney disease stage IIIb, and coronary artery disease s/p stent placement in the second obtuse marginal branch of the circumflex artery presented to the emergency department with dyspnea on exertion and an unintentional 30-pound weight loss over two to three months. His prior occupation included work in a tire factory; he denied exposure to asbestos. He chewed tobacco and had a dog. A chest radiograph showed a large right-sided pleural effusion. He had undergone repeated thoracentesis and pigtail chest tube placements over four hospital admissions.

Review of the initial cytology, extensive serology, pleural fluid analysis, Gram stain, culture, HIV testing, and adenosine deaminase testing was negative. Given the recurrent hospitalizations and reaccumulation of the right pleural effusion, the decision was made to proceed with endobronchial ultrasound, which demonstrated lymphocytosis without evidence of malignancy. Bronchoalveolar lavage grew 100 colonies of *Cryptococcus neoformans*. The patient underwent lumbar puncture with an opening pressure of 37 and a negative cryptococcal antigen. He was started on fluconazole 400 mg daily upon discharge, with a suspected treatment course of 12 months and close outpatient pulmonology follow-up. This report highlights a rare initial presentation of *Cryptococcus neoformans *manifesting as recurrent unilateral pleural effusion.

## Introduction

*Cryptococcus neoformans* infection presenting as a rapidly reaccumulating unilateral pleural effusion in a non-immunocompromised host is a rare presentation of pulmonary cryptococcal disease. While the usual presentation is meningitis in immunocompromised patients, we present a rare case of recurrent unilateral pleural effusion as the initial presentation of cryptococcal disease in an immunocompetent patient.

Cryptococcal pneumonia is an opportunistic fungal infection commonly transmitted through inhalation of the fungus into the lungs. There is an association with exposure to pigeons and their droppings contaminated with the fungus [1,2]. Infection is more common in immunosuppressed individuals, including those with a history of HIV, AIDS, cirrhosis, or those receiving immunosuppressive therapies [3]. In rare cases, infection can occur in immunocompetent hosts. In immunocompetent hosts, cryptococcal pneumonia is a common presentation and may occur asymptomatically or with nonspecific symptoms, including cough, chest pain, sputum production, weight loss, and fever [1].

Common radiological findings associated with cryptococcal pneumonia include alveolar infiltration and consolidation, lymphadenopathy, and, more rarely in immunocompetent hosts, pleural effusion and cavitation [1]. From the lungs, the infection can spread hematogenously to the brain or meninges, resulting in disseminated disease, which is more commonly seen in immunosuppressed hosts. The presentation of disseminated cryptococcal disease can be similar in immunocompetent hosts, with or without findings suggestive of CNS involvement, such as altered mental status, meningismus, photophobia, or focal neurological findings. In these patients, radiological findings such as lymphadenopathy, pleural effusion, and cavitation are more commonly observed.

Definitive diagnosis of cryptococcal infection is made through tissue biopsy and CSF culture [1]. Other diagnostic tools include sputum culture, bronchoalveolar lavage, serum cryptococcal antigen titers, and lumbar puncture. Management of pulmonary cryptococcal infection in immunocompetent hosts includes ketoconazole at a dose of 200-800 mg/day [1]. To our knowledge, there have been fewer than six cases of pulmonary cryptococcal infection presenting as unilateral pleural effusion [4,5]. Even rarer is a presentation of cryptococcal pneumonia without the most common radiologic finding of pulmonary nodules [6]. This report describes a rare initial presentation of *Cryptococcus neoformans* infection manifesting as recurrent unilateral pleural effusion.

This case report was previously presented as a poster at the 2026 ATS International Conference on May 17, 2026.

## Case presentation

An 85-year-old male with a past medical history of hypertension, hyperlipidemia, diabetes, chronic kidney disease stage IIIb, and coronary artery disease status post stent placement in the second obtuse marginal branch of the circumflex artery presented to the emergency department with dyspnea on exertion and an unintentional 30-pound weight loss over two to three months. His prior occupation included work in a tire factory, although he denied exposure to asbestos. He was widowed, lived alone with his dog, and reported chewing tobacco use. Before his initial hospitalization, the patient had been evaluated by his PCP, diagnosed with pneumonia, and started on antibiotics. The patient then presented to the ED with progressive dyspnea and a nonproductive cough of two weeks' duration. ED labs were significant for a proBNP level of 5200, renal function near baseline, and a CBC without leukocytosis.

Chest X-ray demonstrated a new large right pleural effusion, and the EKG showed new-onset atrial fibrillation. He was continued on empiric antibiotics, including vancomycin and Zosyn, due to failure of outpatient therapy. He underwent thoracentesis, with 1650 mL of amber fluid drained from the right chest, resulting in improvement of symptoms. Pleural fluid was sent for analysis. CT chest with contrast was performed for further evaluation of pulmonary processes and demonstrated no lesions suspicious for malignancy. Echocardiogram demonstrated an ejection fraction of 55-60%, and he remained asymptomatic from the new-onset atrial fibrillation. TSH was within normal limits. He was discharged after three days with improvement in symptoms. Antibiotics were discontinued due to no indication for continued therapy, and he was started on Eliquis for new-onset atrial fibrillation. Pleural fluid studies were consistent with exudative fluid, demonstrated 22% lymphocytes, were negative for metastatic disease, and culture was negative.

Seven days later, the patient again presented to the ED for worsening shortness of breath. He had seen his PCP that same day and was encouraged to go to the ED for recurrent right-sided pleural effusion. He reported associated symptoms of chest pain, nausea, poor appetite, and a productive cough with white phlegm. Additionally, the patient reported a 27-pound weight loss over the past month. ED labs were significant for no leukocytosis, acute kidney injury (AKI; Cr 3.77 mg/dL on admission compared with 1.96 mg/dL at discharge), and elevated proBNP. CXR demonstrated a persistent large right pleural effusion (Figure 1).

**Figure 1:**
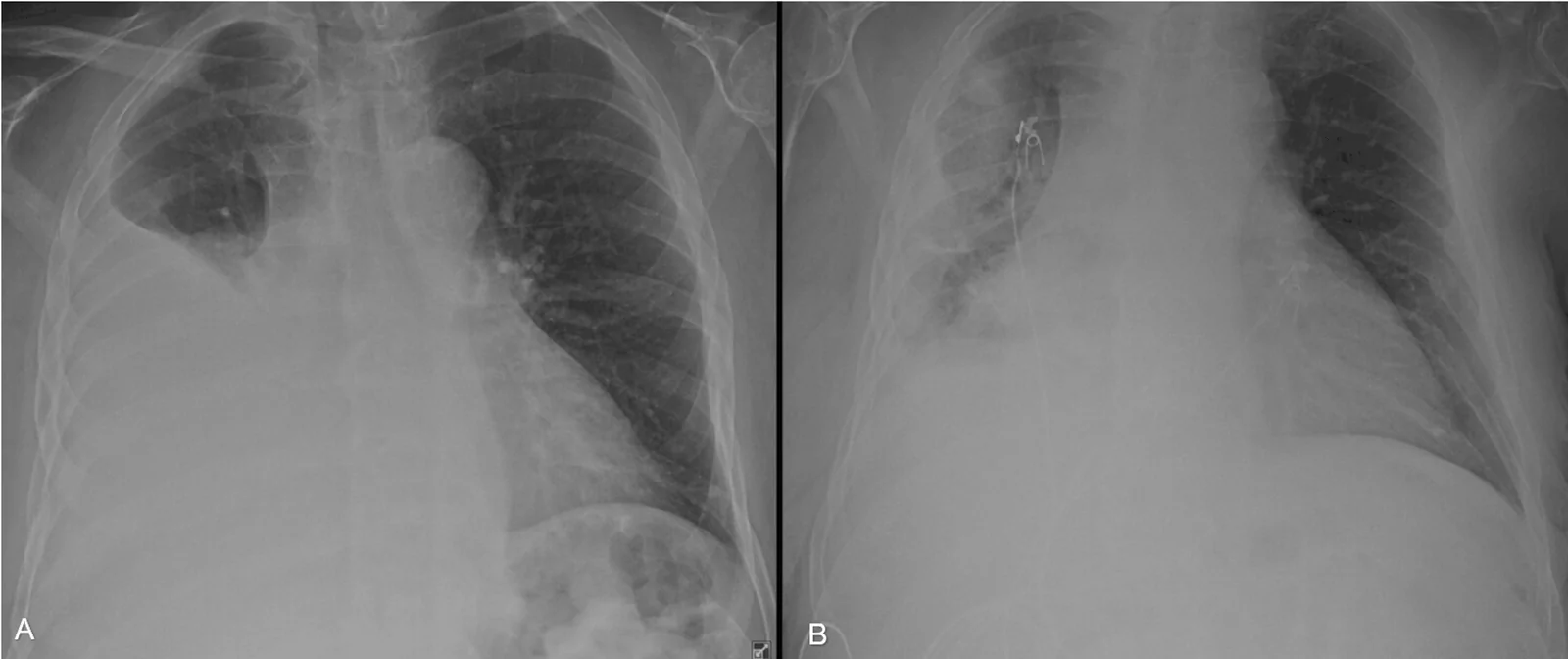
Chest X-ray demonstrating the persistence of a large right pleural effusion A: First admission. B: Second admission (seven days later)

At this point, malignancy versus TB or other infectious etiologies could not be excluded. Thoracentesis was performed, with 1860 mL of serous fluid drained from the right chest. Urine sodium and urine creatinine were ordered to evaluate the etiology of AKI; urine sodium was 82 mEq/L and urine creatinine was 42.8 mg/dL, with a fractional excretion of sodium of 5.3%, suggesting an intrinsic cause of AKI. A repeat CT chest with contrast was not performed due to AKI; CT chest without contrast demonstrated a persistent large right-sided effusion. Repeat echocardiogram demonstrated an ejection fraction of 55-60%, right ventricular systolic pressure of 34, and no diastolic dysfunction. A second thoracentesis was performed during this hospitalization, with 1.4 L of serosanguineous fluid removed and sent for cytology.

Fluid analyses were consistent with an exudative and monocyte-predominant process, with adenosine deaminase levels not consistent with TB, and cytology was negative. The pleural effusion did not rapidly reaccumulate over five days during admission. The patient was discharged after eight days in stable condition, status post two thoracenteses, with cytology negative for malignancy twice and TB ruled out. He was advised to follow up with pulmonology in six weeks, with consideration of lung biopsy or PleurX catheter placement as potential next steps if clinically indicated.

The patient presented to the ED 14 days later with the same complaint of shortness of breath. He endorsed chest pain and increased shortness of breath. ED labs were significant for leukopenia of 3.4. CT chest demonstrated a large right pleural effusion that had grown in size compared to previous imaging (Figure 2).

**Figure 2:**
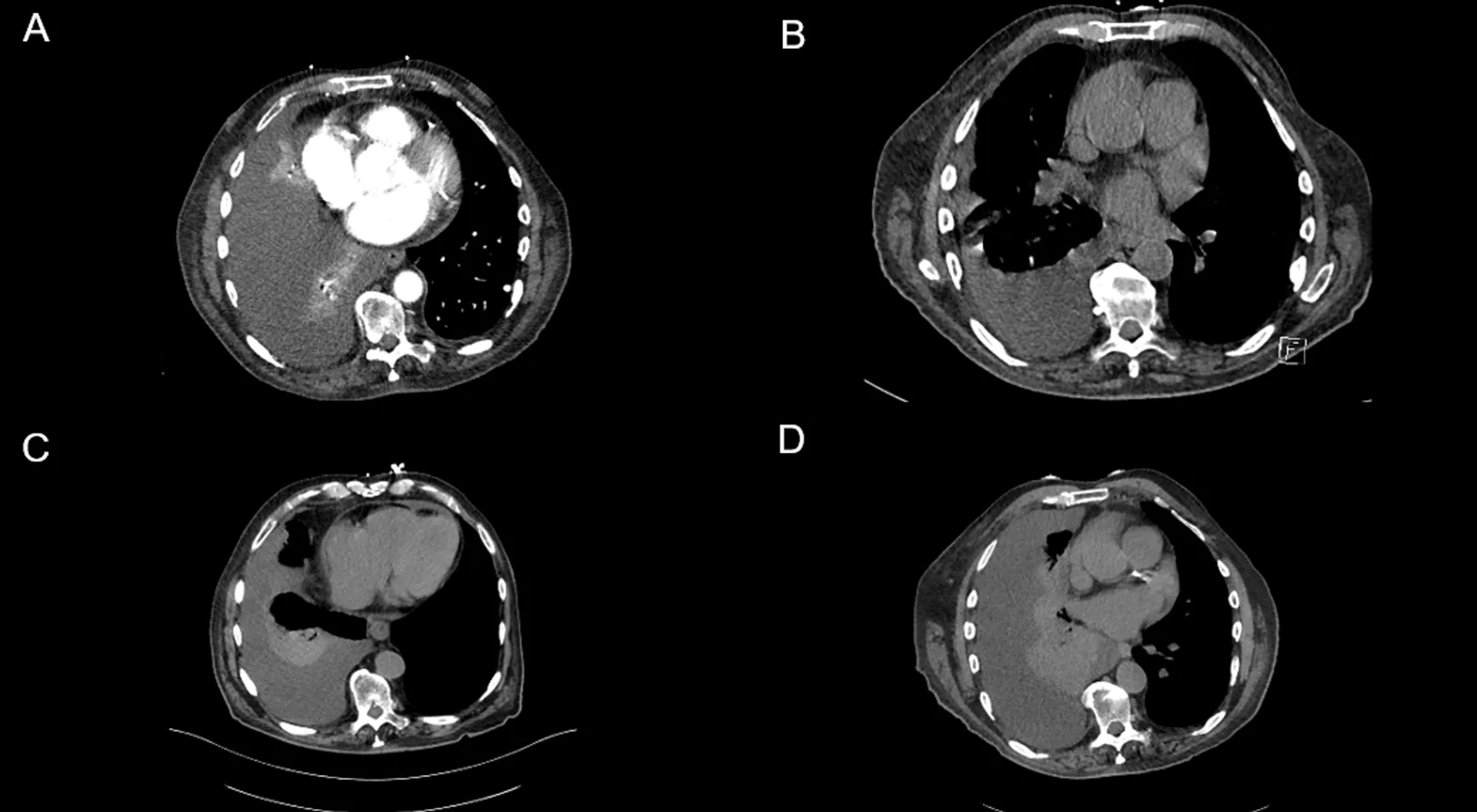
CT findings The image shows serial reaccumulation of the unilateral pleural effusion causing readmission. Starting from the initial admission (day one) to the fourth admission (day 27), as seen on CT of the abdomen and pelvis on day one (A), day eight (B), and day nine (C), and CT of the chest on day 27 (D) CT: computed tomography

A pigtail catheter chest tube was placed in the ED with immediate output of 1300 mL of straw-colored fluid, with daily output remaining >150 mL. Pleural cytology was negative, and flow cytometry was not consistent with malignancy. CT of the chest, abdomen, and pelvis was performed to evaluate for underlying malignancy and demonstrated no defined mass lesion, although heterogeneous airspace opacities could obscure a mass lesion, and prominent mediastinal and hilar lymph nodes were indeterminate for reactive versus malignant etiology. Bronchoscopy and endobronchial ultrasound with mediastinal lymph node sampling were performed, with specimens obtained from brushing of the right middle lobe lateral segment, bronchoalveolar lavage of the right middle lobe lateral segment, and stations 4R and 12R. Rapid on-site evaluation demonstrated lymphocytes and no evidence of malignancy.

At this point, the etiology was unclear. The plan was for discharge with outpatient pulmonology follow-up for PleurX catheter placement. During the outpatient pulmonology visit, the bronchoalveolar lavage was positive for 100 colony-forming units per mL of *Cryptococcus neoformans* (Table 1). Upon receipt of these results, the patient was directly admitted to the hospital. Lumbar puncture was performed, and cryptococcal antigen was negative, ruling out cryptococcal meningitis. HSV and HIV serology were negative. The patient was discharged with a recommendation from Infectious Disease for fluconazole 400 mg daily for 6-12 months, with outpatient pulmonology follow-up.

**Table 1:** Serology, pleural fluid, and BAL studies with evidence of pulmonary Cryptococcus neoformans BAL: bronchoalveolar lavage; SARS-CoV-2: severe acute respiratory syndrome coronavirus 2; CSF: cerebrospinal fluid; CFU: colony-forming unit

Variable	Patient value	Normal value
Aspergillus flavus	0.18	< 0.50
Fungitell	< 45 pg/mL	< 60 pg/mL
Cryptococcal antigen	Negative	Negative
HIV	Negative	Negative
Quantiferon-TB	Negative	Negative
Influenzae A	Negative	Negative
Influenzae B	Negative	Negative
SARS-CoV-2	Negative	Negative
Cryptococcal Ag CSF	Negative	Negative
Pleural fluid: Gram stain	No organisms	No organisms
Pleural fluid: fungal stain	No organisms	No organisms
Adenosine deaminase	Negative	Negative
Cryptococcus neoformans BAL	100 CFU	0 CFU
Aspergillus Ag BAL	Not detected	Not detected
Actinomyces BAL	1 CFU	< 100 CFU

## Discussion

Pulmonary *Cryptococcus neoformans* infection is a rare presentation of cryptococcosis among immunocompetent hosts. The case presented in this report demonstrates this rare presentation. This patient presented without a recent travel or exposure history. The patient did not have HIV, AIDS, or cirrhosis (Table 1). Common risk factors for cryptococcal infection include HIV, AIDS, cirrhosis, and the use of immunosuppressive therapies [3]. Our patient did have a history of diabetes mellitus, which is a common underlying disease in pulmonary cryptococcal disease [7]. Typical exposure is through bird droppings and soil colonization; our patient likely had exposure via inhalation of spores from the environment, with direct fungal infection of the parenchyma with visceral pleural involvement, resulting in a localized infection rather than disseminated disease with central nervous system involvement. His initial chief complaint of pleural effusion was an uncommon presentation.

The most common causes of pleural effusion include congestive heart failure, pulmonary infection, and malignancy [8]. Our patient had no history of heart failure, initially had no evidence of an underlying infection, and had no evidence of malignancy on laboratory workup. Radiological findings in immunocompetent patients rarely include areas of hazy increased attenuation, airspace consolidation, pleural effusion, or adenopathy [9,10]. CT findings of mediastinal lymphadenopathy and cavitary lesions are often more prominent in the immunocompromised patient population [11]. While symptoms are often nonspecific, such as dyspnea, there were no significant differences in symptom presentation between immunocompetent and immunocompromised patients. The patient's symptoms overlapped between disseminated and pulmonary cryptococcal disease. One retrospective study found that pleural effusion was more common in disseminated cryptococcal disease and was associated with poorer outcomes [12]. This patient was subsequently discharged with appropriate antifungal treatment and close outpatient pulmonary follow-up.

## Conclusions

This report discusses a rare initial presentation of pulmonary cryptococcal infection as a recurrent unilateral pleural effusion. Cryptococcal infection should be included in the differential diagnosis in both immunocompetent and immunocompromised patients presenting with pleural effusion. Evaluation should include a thorough history and physical examination, laboratory and imaging workup, bronchoalveolar lavage, lumbar puncture, and/or pulmonary biopsy to differentiate isolated pulmonary disease from disseminated disease.
